# An analysis of p53, BAX and vascular endothelial growth factor expression in node-positive rectal cancer. Relationships with tumour recurrence and event-free survival of patients treated with adjuvant chemoradiation

**DOI:** 10.1038/sj.bjc.6600155

**Published:** 2002-03-04

**Authors:** S Cascinu, F Graziano, V Catalano, M P Staccioli, M C Rossi, A M Baldelli, S Barni, A Brenna, S Secondino, P Muretto, G Catalano

**Affiliations:** Division of Medical Oncology, Hospital of Parma, Italy; Medical Oncology Unit, Hospital of Urbino, Italy; Division of Medical Oncology, Hospital of Pesaro, Italy; Department of Histopathology, Hospital of Pesaro, Italy; Division of Medical Oncology, Hospital of Treviglio, Italy; Department of Histopathology, Hospital of Monza, Italy

**Keywords:** rectal cancer, apoptosis, angiogenesis, metastasis, chemotherapy, radiotherapy, p53, BAX, vascular endothelial growth factor

## Abstract

Tumours of patients with node-positive rectal cancer were studied by immunohistochemistry for p53, BAX and vascular endothelial growth factor expressions. Results were correlated to the relapse rate, the pattern of relapse and the event-free survival after radical surgery and adjuvant chemoradiation. After a median follow-up of 60 months, 39 patients remained disease-free and 40 patients relapsed (18 local relapses and 22 distant metastases). The majority of disease-free patients showed p53 negative and vascular endothelial growth factor negative tumours. Local relapses occurred more frequently in patients with p53 overexpressing tumours (*P*<0.01), while distant metastases were in patients with vascular endothelial growth factor positive tumours (*P*<0.003). Patients with p53 negative or vascular endothelial growth factor negative tumours showed better event-free survival than patients with p53 positive or vascular endothelial growth factor positive tumours. BAX analysis did not show any association with patients' outcome and it was unrelated to the p53 status. Adjuvant treatment strategies for node-positive rectal cancer may be improved by identifying categories of high-risk patients. In this study, vascular endothelial growth factor and p53 expressions correlated with recurrent disease, pattern of relapse and poor event-free survival.

*British Journal of Cancer* (2002) **86**, 744–749. DOI: 10.1038/sj/bjc/6600155 www.bjcancer.com

© 2002 Cancer Research UK

## 

Adjuvant chemoradiation represents the standard of care for patients with resected stage II and III rectal cancer (Minsky, 1999). However, in spite of adjuvant therapies, a significant proportion of patients show local or distant recurrences with detrimental effect on quality of life and survival. The knowledge of molecular features which determine the behaviour of individual tumours may represent a fundamental step to identify high-risk categories of patients and optimize therapeutic strategies (Vaughn and Haller, 1997).

So far, the tumour-node-metastasis (TNM) staging system has been considered the most important independent prognostic feature in primary resected rectal cancer, however, it seems insufficient to identify subsets of prognostic categories. Recent investigations have focused on innovative molecular markers which may explain differences in the outcome of colorectal cancer patients belonging to a homogenous TNM stage group (McLeod and Murray, 1999).

Angiogenesis represents a key event in the process of tumour invasion and metastasis (Folkman, 1996) and the vascular endothelial growth factor (VEGF) is one of the most important molecules promoting endothelial cell migration, proliferation and differentiation (Grunstein *et al*, 1999; Arii *et al*, 1999). The expression of this glycoprotein has been investigated in colorectal carcinomas and its up-regulation has been related to the occurrence of relapses and the poor prognosis of primary resected colorectal cancer (Takahashi *et al*, 1995; Ishigami *et al*, 1998; Cascinu *et al*, 2000, 2001).

The ability of radiation and chemotherapy to eradicate tumour cells and prevent relapses depends on successful induction of apoptosis in response to DNA damage. The fine interplay between the Bcl-2 family anti-apoptotic members and death-promoting members like BAX and p53 ensures a regular apoptotic process, while its disruption causes the loss of programmed cell death (Reed, 1999).

The BAX protein plays a central role in regulating apoptosis (Yin *et al*, 1997). It is located in the outer mitochondrial membrane and its expression induces mitochondrial permeability which leads to the release of cytochrome-*c* and a downstream cascade of events promoting DNA degradation and cell death. In experimental models, the p53 protein was found to be a positive regulator of BAX transcription, also, the analysis of the p53/BAX apoptotic pathway in colorectal carcinomas showed potential prognostic value (Zhan *et al*, 1994; Miyashita and Reed, 1995; De Angelis *et al*, 1998; Simms *et al*, 1998; Sturm *et al*, 1999).

These data prompted us to investigate markers of angiogenesis/apoptosis in rectal carcinomas of patients with resected, node-positive disease. In their tumours, we evaluated the VEGF expression and the p53/BAX apoptotic pathway; these molecular features were related to the event-free survival, the relapse rate and the patterns of recurrences after adjuvant chemoradiation.

## MATERIALS AND METHODS

### Human samples and clinicopathologic data

In this retrospective analysis, the study population consisted of consecutive patients who underwent curative surgery for stage III rectal cancer between 1994 and 1996. Inclusion criteria were: available archival tissue of the primary tumour, radical surgery with negative resection margins and adequate follow-up information (patients had to be observed for 5 years after surgery at least). For the purpose of the analysis, patients had to be treated with a homogeneous adjuvant protocol based on six chemotherapy cycles with bolus fluorouracil/folinic and pelvic radiation 45 Gy, with a boost to a total of 54 Gy.

The follow-up consisted of interim history, physical examination, haematologic studies, carcinoembryonic antigen levels and diagnostic imaging (chest X-ray, abdominal ultrasonography) every 4 months in the first year, and every 6 months in the second through fifth years. Patients had barium enema or a colonoscopic examination 6 months after surgery and subsequently every 12 months. Abdominal and pelvic computed tomographic scan was performed for corroborative evidence of relapse. The recurrences of rectal carcinoma had to be proven by cytology biopsy or surgery.

The analysis was carried out on the primary tumour and all the cases were reviewed by the pathologist. The selected blocks were those in which mucosa, invasive edge and viable tumour were present. The study was performed in a blind fashion, so that patients' outcome was unknown by the investigators performing VEGF, p53 and BAX measurements.

### BAX and p53 analyses

Formalin-fixed, paraffin-embedded tumour blocks were analysed immunohistochemically for BAX and p53 expressions using a standard avidin-biotin technique. Commercially available antibodies which have been widely used for prognostic investigations in colorectal cancer were chosen for the analysis (Baas *et al*, 1994; McLeod and Murray, 1999; Sturm *et al*, 1999).

Sections (4 μm thick) were deparaffinized in xylene, rehydratated in graded ethanol series and incubated in 3% hydrogen-peroxide for 20 min. Specimens were placed in a plastic Coplin jar with citric buffer and heated 4×2.5 min in a microwave processor at 95°C. Subsequently, sections were left in a Coplin jar at room temperature for 30 min. Specimens were covered with normal goat serum for 15 min to reduce nonspecific staining and incubated at room temperature for 1 h with murine p53 monoclonal antibody (DAKO DO7, Copenhagen, Denmark; dilution 1 : 75) (Baas *et al*, 1994) and rabbit polyclonal antibody for BAX (Oncogene Research Products, Cambridge MA, USA; dilution 1 : 50) (Sturm *et al*, 1999). Sections were washed with Tris-buffered saline (TBS), incubated with 1 : 100 dilution of biotinylated goat anti-mouse IgG at room temperature for 30 min, and covered with 1 : 100 dilution of streptavidin-biotin-peroxidase complex at room temperature for 30 min. The antibody was localized with 3,3′-diaminobenzidine tetrahydrochloride. Tissue sections were counterstained with light haematoxylin, dehydrated with ethanol and mounted under a coverslip. Positive control sections were from a colon carcinoma known to express high p53 and BAX proteins levels. TBS, instead of the primary antibody was used as negative control.

In each case, the entire section was examined on high-power fields (×400) for BAX (cytoplasmic) and p53 (nuclear) immunoreactivity. The level of immunoreactivity was expressed as percentage of stained cancer cells (0 to 100%). For the purpose of the study and based on previous experiences (Sturm *et al*, 1999; Schwander *et al*, 2000a), BAX and p53 expressions were classified as negative (none or ⩽10% of tumour cells stained) and positive (>10% of tumour cells stained).

### VEGF analysis

Sections (4 μm thick) of the primary tumour tissue were deparaffined in xylene and rehydrated in a graded ethanol series. Specimens were placed in a Coplin jar containing citric buffer and heated 3×5 min in a microwave processor. Subsequently, the sections were left in the Coplin jar at room temperature for 20 min. After an incubation in 3% hydrogen-peroxide for 8 min, specimens were covered with normal swine serum for 10 min to reduce nonspecific staining and incubated with a 1 : 20 dilution of rabbit polyclonal antibody for VEGF (Biogenex, San Ramon, CA, USA) at room temperature for 30 min. The sections were washed and incubated with a 1 : 50 dilution of biotinylated swine anti-rabbit IgG at room temperature for 20 min and then covered with a 1 : 100 dilution of streptavidin-biotin-peroxidase complex at room temperature for 20 min. The antibody was localized with 3,3′-diaminobenzidine tetrahydrochloride. Tissue sections were counterstained with light haematoxylin, dehydrated with ethanol and mounted under a coverslip. Normal rabbit IgG was substituted for primary antibody as the negative control. For positive controls, normal mucosa known to express VEGF was stained for VEGF.

The staining results were evaluated independently by two investigators and each entire section was examined on high-power fields. VEGF expression showed cytoplasmic localization and only clearly immunoreactive cells were recorded positive. VEGF expression was scored as percentage of immunoreactive cells and each case was categorized as positive (>10% of tumour cells stained) or negative (none or ⩽10% of tumour cells stained) (Cascinu *et al*, 2000, 2001).

### Statistical analysis

Statistical analysis was performed to correlate the results of BAX, p53, VEGF to the event-free survival and to the relapse rate after adjuvant chemoradiation. Also, the pattern of relapse (local vs distant metastases) was studied in relation to the expression of the three markers. According to the cut-off values, the results of BAX, p53 and VEGF analyses were used as dichotomized (categorical) variable. Contingency tables were analysed by the Fisher's exact test or the Chi-square test as appropriate. The event-free survival was calculated from the data of surgery to the time of confirmed relapse and the Kaplan–Meier method was adopted to estimate survival curves. Differences between survival curves were studied by means of the log-rank test. In addition, the proportional hazards model (Cox, 1972) was used to assess the prognostic importance of the three biomarkers for event-free survival adjusting for relevant baseline clinico-pathologic features (Moran *et al*, 1992; Stocchi *et al*, 2001). All the values were two-sided and statistical significance was defined as *P*<0.05.

## RESULTS

### Characteristics of patients

According to the inclusion criteria, tumours of 79 out of 87 consecutive patients were analysed. Eight cases were excluded (9%); five because of insufficient follow-up information and four due to unassessable archival tissue. In the 79 assessable patients, 39 patients remained disease-free, 18 patients had local relapse and 22 patients showed distant metastases. The distribution of clinicopathologic variables and the outcome after surgery and adjuvant chemoradiation are reported in Table 1

**Table 1:**
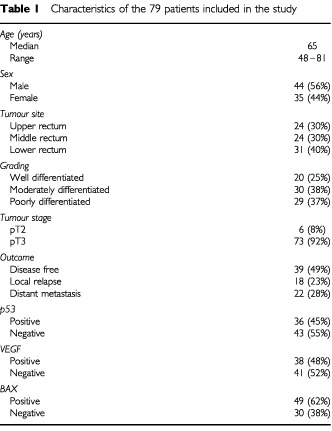
Characteristics of the 79 patients included in the study

.

### BAX and p53 analysis

**Figure 1:**
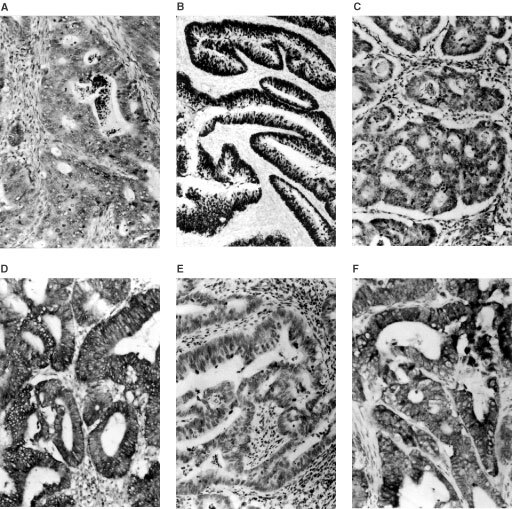
Examples of p53, BAX and VEGF immunohistochemical analyses in specimens of rectal carcinomas. Negative (**A**) and positive (**B**) p53 nuclear immunoreactivity. Negative (**C**) and positive (**D**) cytoplasmic VEGF immunoreactivity. Negative (**E**) and positive (**F**) cytoplasmic BAX immunoreactivity.

Examples of immunohistochemical analysis for p53 and BAX proteins expression are reported in Figure 1

. High cytoplasmic BAX protein levels were detected in 49 cases (62%) and low expression in the remaining 30 cases (38%). The p53 protein was overexpressed in 36 cases (45%) and it was low or undetectable in 43 cases (55%). A preliminary combined analysis of BAX and p53 expressions did not show any association between the two markers (data not shown).

The distribution of BAX positive and negative cases in disease-free and relapsed patients did not show any significant correlation (Table 2

**Table 2:**
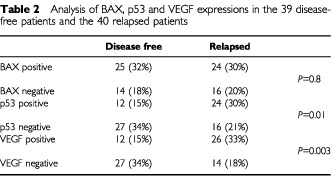
Analysis of BAX, p53 and VEGF expressions in the 39 disease-free patients and the 40 relapsed patients

). Also, differences between event-free survival curves of patients with BAX positive and BAX negative tumours were not statistically significant. Patients whose tumours showed p53 overexpression had higher frequency of recurrences (Table 2) and worse event-free survival (*P*<0.01) than patients with with p53 negative tumours (Figure 2A

**Figure 2:**
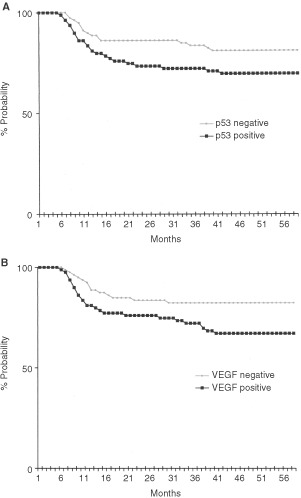
(**A**) Event-free survival analysis in the 36 patients with p53 overexpressing tumours and the 43 patients with p53 negative tumours. (**B**) Event-free survival analysis in the 38 patients with VEGF positive tumours and the 41 patients with VEGF negative tumours.

).

### VEGF analysis

An example of immunohistochemical analysis for VEGF expression is reported in Figure 1. VEGF positive and negative cases were 38 (48%) and 41 (52%) respectively. A significantly higher proportion of relapsed patients had VEGF positive tumours (26 out of 79 cases; 33%), while the majority of disease-free patients (27 out of 79 cases; 34%) showed VEGF negative tumours (Table 2). Patients with VEGF positive tumours showed worse event-free survival (*P*<0.001) than patients with VEGF positive tumours (Figure 2B).

### Local *vs* distant recurrences

The VEGF expression and the p53 status resulted in the two molecular features which showed a significant correlation with patients' outcome. Accordingly, a further analysis of VEGF and p53 was performed in tumours of the 18 patients with local recurrences and the 22 patients with distant metastases (Table 3

**Table 3:**
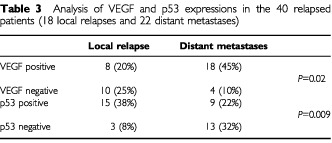
Analysis of VEGF and p53 expressions in the 40 relapsed patients (18 local relapses and 22 distant metastases)

). A significant distribution of expression of both markers was found; the majority of patients with distant metastasis had VEGF positive tumours, on the other hand, local relapses occurred more frequently in patients whose tumours showed p53 overexpression.

### Multivariate analysis

Multivariate analysis with the COX proportional hazards model showed that p53 and VEGF expressions were independent prognostic factors for event-free survival (Table 4

**Table 4:**
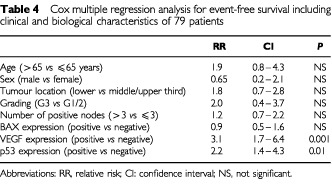
Cox multiple regression analysis for event-free survival including clinical and biological characteristics of 79 patients

), whereas age, sex, grading, tumour site, number of positive lymph nodes and BAX expression were not independent indicators of prognosis.

## DISCUSSION

In colorectal cancer, p53 mutations occur with a frequency of 35 to 60% (Hollstein *et al*, 1991). The most common are missense mutations which usually prolong the half-life of the p53 abnormal protein causing its nuclear accumulation and the detection by immunohistochemistry. In rectal cancer, studies on the prognostic role of p53 showed disappointing results; p53 overexpression or gene mutation were found to have unfavourable influence on survival (Adell *et al*, 1999; Liang *et al*, 1999; Schwadner *et al*, 2000a,b), but other studies did not confirm the relationship between the p53 status and patients' outcome (Elsaleh *et al*, 1999; Nehls *et al*, 1999; Sturm *et al*, 1999).

A positive immunostaining for cytoplasmic BAX protein is an indicator of its preserved pro-apoptotic function and high BAX protein expression was found to possess positive prognostic value in colorectal cancer (Ogura *et al*, 1999; Sturm *et al*, 1999).

Experimental data support a functional relationship between p53 and BAX, in fact, p53 is known to be a transcriptional regulator of the BAX gene and the p53-to-BAX is considered as a major apoptotic pathway (Zhan *et al*, 1994; Miyashita and Reed, 1995; Simms *et al*, 1998). *In vivo*, mechanisms regulating the BAX/p53 apoptotic pathway seem to be more complex and the relationship between the p53 genotype/phenotype and BAX expression was not confirmed in colorectal carcinomas (De Angelis *et al*, 1998). In advanced colorectal cancer, Sturm *et al* (1999) did not find any significant association between BAX expression levels and the p53 status. Schwandner *et al* (2000b) investigated the prognostic value of the apoptotic index compared to molecular features of rectal carcinomas and they found that apoptosis did not possess a prognostic role, whereas p53 was an independent predictor for both recurrence and survival.

A key point for the interpretation of p53 and BAX results are the molecular modifications in response to chemoradiation (Rosen *et al*, 1999). Apoptosis increases after treatment with 5-fluorouracil or radiotherapy and it is correlated with enhanced BAX expression (Sugamura *et al*, 1997; Ohno *et al*, 1998; Kokawa *et al*, 1999). In rectal cancer a significantly higher expression of BAX was observed after preoperative chemoradiation (Tannapfel *et al*, 1998) and in cervical cancer increased BAX expression after radiotherapy was related to better tumour control (Harima *et al*, 2000). These data suggest that raising of BAX expression rather than its basal level may correlate with apoptosis. Accordingly, it is possible that a ‘dynamic’ study of BAX with pre- and post-treatment determinations could clarify interactions with the p53 status and its prognostic role.

In the present study, BAX and p53 expressions did not show any association and only p53 overexpression correlated with local failure after surgery and adjuvant chemoradiation. The predictive value of p53 is supported by experimental data which showed functional relationships between wild-type p53 and radiosensitivity (Rosen *et al*, 1999). In rectal cancer, p53 expression was found to be predictive of response to preoperative chemoradiation (Spitz *et al*, 1997; Fu *et al*, 1998; Luna-Perez *et al*, 1998) and the p53 status correlated with the frequency of local recurrences (Sato *et al*, 1998; Adell *et al*, 1999). Our results seem to confirm the potential predictive role of p53, however, this finding requires further investigation, including a combined analysis with a surgery-alone group.

VEGF up-regulation has been linked to prognosis in colorectal cancer (Takahashi *et al*, 1995; Ishigami *et al*, 1998; Cascinu *et al*, 2000, 2001). In our study, VEGF expression was associated with tumour recurrence and poor event-free survival, also, patients whose tumours were VEGF positive had significantly higher frequency of distant metastases. These data support the role of an angiogenic phenotype in the progression of rectal cancer and the metastatic pattern of individual tumours. Neovascularization sustained by VEGF up-regulation is necessary for tumour nourishment and it is a potential route for haematogenous spread and metastasis. Previous data contribute to support this hypothesis; high VEGF protein expression or mRNA levels correlated with the M1 stage and liver metastases from colorectal carcinomas (Kang *et al*, 1997; Tokunaga *et al*, 1998; Lee *et al*, 2000).

The results of the present study have strengths and limitations. To the best of our knowledge it is the first report of a concomitant analysis for p53, BAX and VEGF expressions in rectal cancer. The analysis has been performed in a homogenous population of patients with node-positive disease whose rates of local and distant metastases are comparable to that of other series (Minsky, 1999). Patients received the same protocol of adjuvant chemoradiation and they underwent fixed follow-up controls. Possible biases could derive from the retrospective nature of the study and the immunohistochemical analysis for p53 which may not always discriminate between wild-type and mutated gene. In colorectal tumours, the 5% p53-positive nuclei was experimentally determined as a relevant cut-off level to assess TP53 gene damage (Clausen *et al*, 1998). However, according to previous prognostic studies in rectal cancer (Sturm *et al*, 1999; Schwander *et al*, 2000a), we set the cut-off value for p53 at 10% positive stained cells. Finally, pre- and post-treatment determinations of BAX could have supplied more information, however, this dynamic analysis was unfeasible in patients who had received postoperative chemoradiation.

In the present study, p53 overexpression and VEGF up-regulation showed a prognostic role and they influenced event-free survival of patients with surgically-resected node-positive rectal cancer. This finding, together with the distinct pattern of relapse associated with p53 overexpression (local recurrences) or VEGF up-regulation (distant metastases) may contribute to identify categories of high-risk patients and tailor specific treatment strategies. More specific and aggressive radiotherapy programs may be delivered to patients with increased risk for local relapses, whereas patients at risk for distant metastases may benefit from more potent combination chemotherapy regimens or new anti-angiogenetic molecules. Our results deserve further investigation, since new prognostic molecular markers may represent a fundamental step to improve the post-surgical management of rectal cancer.
